# Sperm epigenomics: challenges and opportunities

**DOI:** 10.3389/fgene.2014.00330

**Published:** 2014-09-18

**Authors:** Eduard Casas, Tanya Vavouri

**Affiliations:** Institute of Predictive and Personalized Medicine of CancerBarcelona, Spain

**Keywords:** sperm, epigenomics, transcriptome, DNA methylation, chromatin, epigenetic inheritance

## Abstract

Sperm is a highly differentiated cell type whose function is to deliver a haploid genome to the oocyte. The sperm “epigenomes” were traditionally considered to be insignificant – the sperm is transcriptionally inactive, its genome is packaged in sperm-specific protamine toroids instead of nucleosomes, and its DNA methylation profile is erased immediately post-fertilization. Yet, in recent years there has been an increase in the number of reported cases of apparent epigenetic inheritance through the male germline, suggesting that the sperm epigenome may transmit information between generations. At the same time, technical advances have made the genome-wide profiling of different layers of the sperm epigenome feasible. As a result, a large number of datasets have been recently generated and analyzed with the aim to better understand what non-genetic material is contained within the sperm and whether it has any function post-fertilization. Here, we provide an overview of the current knowledge of the sperm epigenomes as well as the challenges in analysing them and the opportunities in understanding the potential non-genetic carriers of information in sperm.

## INTRODUCTION

**FIGURE 1:**
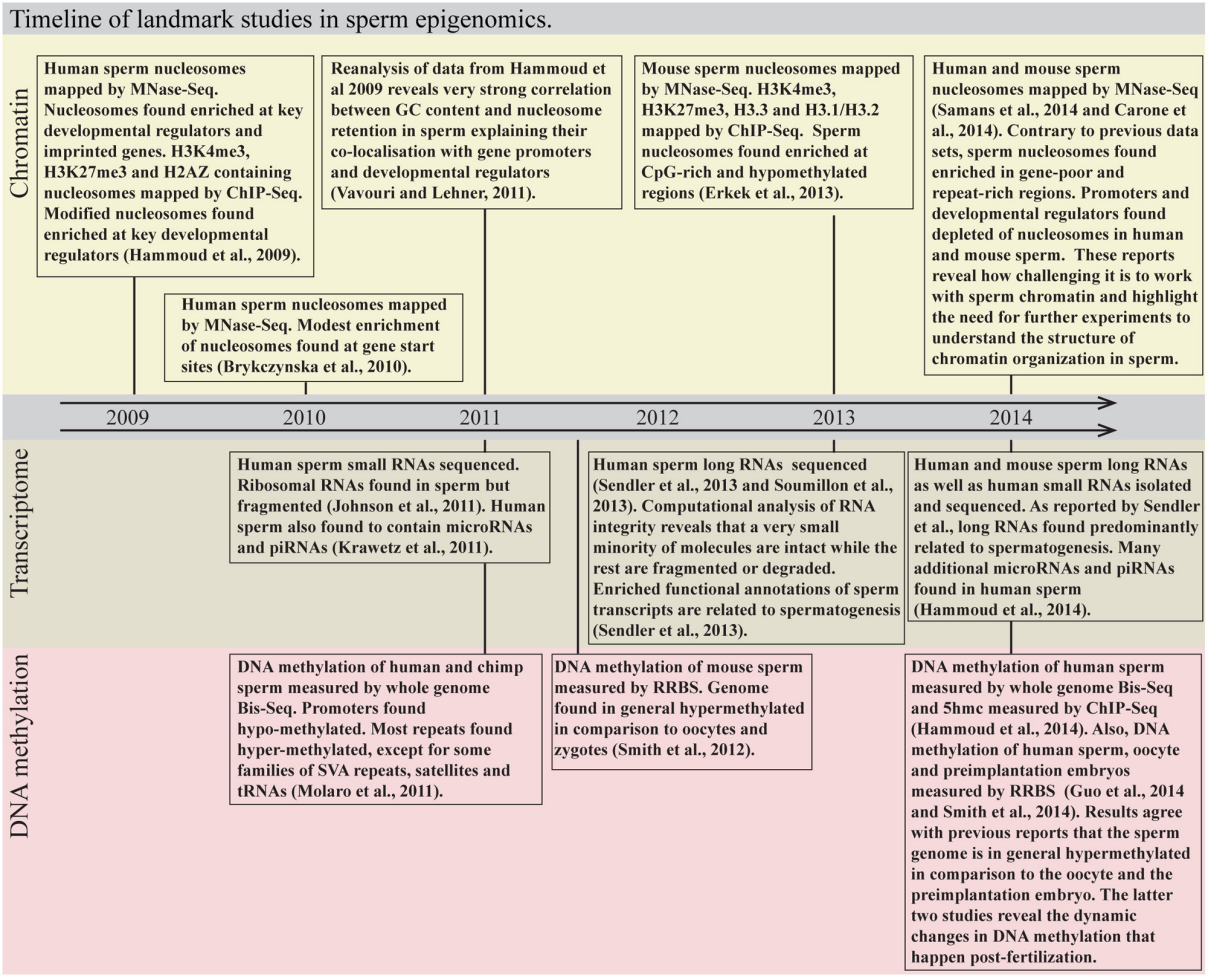
**Timeline of landmark studies in sperm epigenomics.** MNase-Seq, micrococcal nuclease digestion followed by sequencing; ChIP-Seq, chromatin immunoprecipitation followed by sequencing; Bis-Seq, bisulfite sequencing; RRBS, reduced representation bisulfite sequencing.

Sperm are highly specialized cells that propagate genetic material from father to offspring. Animal studies suggest that mammalian sperm can transmit non-genetic information across generations. This epigenetic information may alter depending upon the father’s environmental exposures. In recent years, the different sperm “epigenomes” have been profiled using high throughput sequencing. Sperm is turning from being one of the most poorly to one of the most intensely profiled cell types (**Figure 1**).

Here, we review what is currently known about the RNA, chromatin and DNA methylation profiles of sperm with a focus on human and mouse. We then discuss the experimental and computational challenges in the generation and analysis of sperm epigenome data. Last, we highlight the opportunities raised and the questions that remain unanswered regarding the contents of sperm, especially those related to the impact its non-genetic material has post-fertilization.

## SPERM TRANSCRIPTOME

**FIGURE 2:**
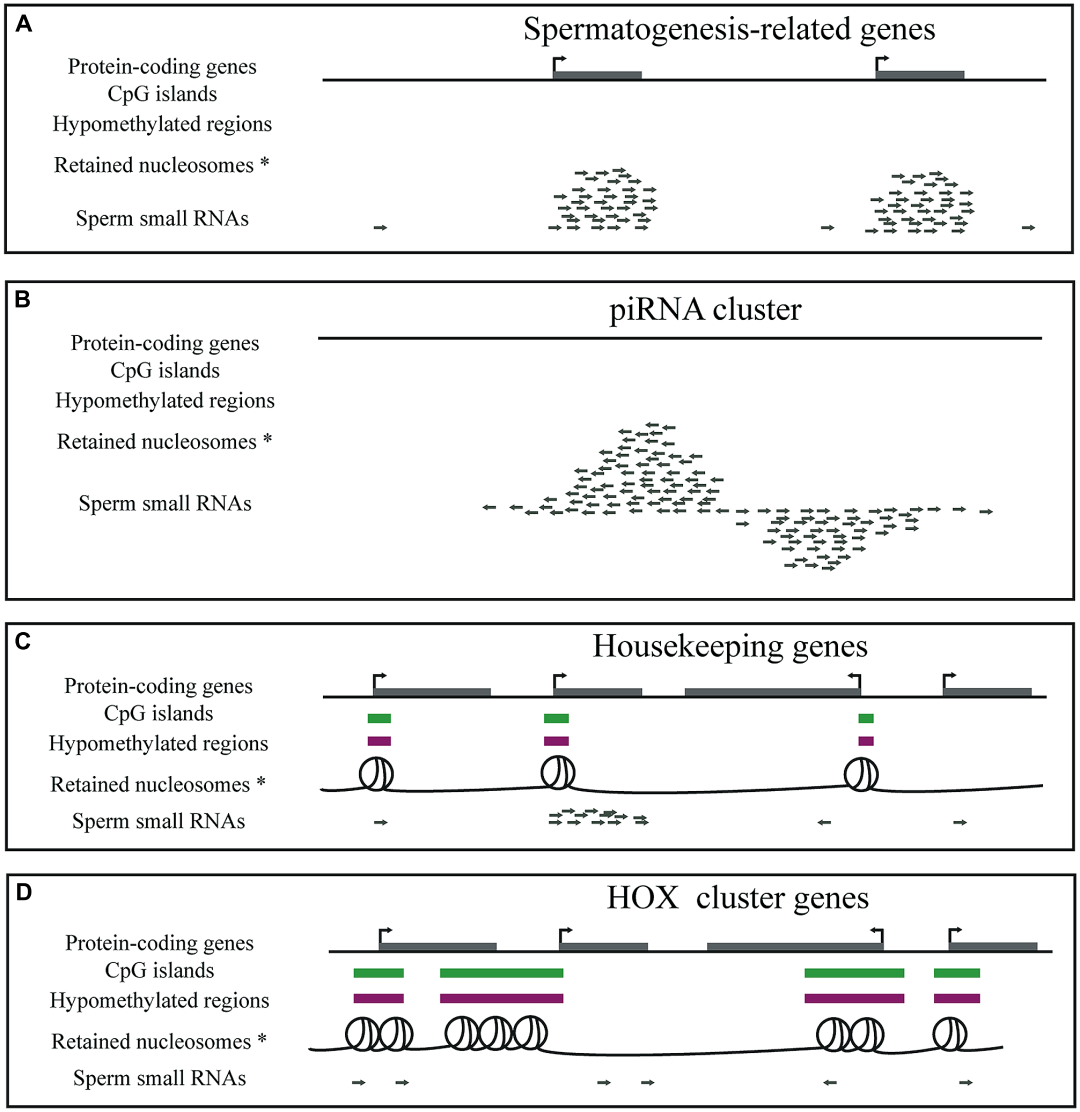
**Diagrammatic representation of genes, CpG islands, DNA methylation, nucleosome retention and small RNAs in mature sperm. (A)** Sperm cells contain a large number of small RNAs that are fragments of spermatogenesis-related genes, such as the protamine genes. **(B)** Sperm cells contain piRNAs. **(C)** GC- and CpG-rich regions overlapping housekeeping gene promoters are hypomethylated and retain nucleosomes in sperm. Small RNA fragments of housekeeping genes expressed until late in sperm development are also present in mature sperm. **(D)** GC- and CpG-rich regions overlapping developmental regulators, such as the HOX cluster genes, are hypomethylated and retain nucleosomes in sperm. *Note that two of the five genome-wide sperm nucleosome datasets claim that nucleosomes are instead depleted from promoters and enriched at gene poor regions.

Mature sperm cells are transcriptionally inactive (Grunewald et al., 2005; Goodrich et al., 2013). Yet, they do contain RNA (Miller et al., 1994). The vast majority of RNA molecules in sperm are fragments of longer transcripts (Johnson et al., 2011; Sendler et al., 2013; Soumillon et al., 2013; **Figure 2A**). This includes ribosomal RNA as well as testes and spermatogenesis-specific mRNAs (Johnson et al., 2011). Cessation of transcription and fragmentation of existing sperm mRNAs may be one of the several safety mechanisms that ensure that, upon fertilization, the highly differentiated sperm gives rise to the totipotent zygote. Sperm transcript fragments are an easily accessible record of transcription of the late stages of sperm differentiation and have the potential to be used as markers of fertility (e.g., Yatsenko et al., 2006; Platts et al., 2007).

In addition to fragments of longer transcripts, sperm cells contain a large repertoire of small non-coding RNAs. Like all other cell types, male germ cells express and require the activity of microRNAs (Hayashi et al., 2008; Maatouk et al., 2008; Romero et al., 2011; Wu et al., 2012) and many can still be detected in mature sperm (Amanai et al., 2006; Krawetz et al., 2011; Hammoud et al., 2014). In comparison to oocytes, sperm appears to make an almost insignificant contribution to the total microRNA content of the zygote (Amanai et al., 2006). Nonetheless, at least two different studies have reported that inhibition, in the zygote, of sperm-delivered microRNAs leads to developmental delays (Liu et al., 2012; Hammoud et al., 2014).

Furthermore, dysregulation of at least two different microRNAs (miR-1 and miR-124) in sperm and their transmission to the egg have been postulated to be the causes of two cases of intergenerational inheritance in mouse (Wagner et al., 2008; Grandjean et al., 2009). It should be noted that similar responses were elicited by microinjections of transcript fragments through an unknown mechanism. Also, it was recently shown that traumatic stress in early life of males alters the sperm microRNA (and PIWI-interacting RNA) profile and behavioral and metabolic responses in the offspring (Gapp et al., 2014). These experiments therefore provide evidence that although sperm contains a small quantity of microRNAs in comparison to the oocyte, it still delivers enough to influence preimplantation development and the phenotype of the offspring.

Male germ cells express PIWI-interacting RNAs (piRNAs; Aravin et al., 2006; Girard et al., 2006; Grivna et al., 2006; Lau et al., 2006; Watanabe et al., 2006), also essential small non-coding RNAs for sperm (Deng and Lin, 2002; Kuramochi-Miyagawa et al., 2004; Reuter et al., 2011; **Figure 2B**). A lot remains to be understood about their function, processing and mechanism of action. Their most deeply conserved function is protection of the germline genome from transposons (reviewed in O’Donnell and Boeke, 2007; Thomson and Lin, 2009; Siomi et al., 2011). piRNAs target transposon transcripts for degradation and silencing when DNA methylation (the “default” mechanism of transposon repression) is nearly completely depleted during germ cell development. In addition, a very small number of piRNAs have been linked to imprinting in mouse (Watanabe et al., 2011). Later in sperm development, the role of piRNAs is not as clear, although there is evidence that piRNAs may still protect the genome from transposons (Di Giacomo et al., 2013). Although initially thought to be absent from mature spermatozoa, recent small RNA sequencing studies have revealed more than a thousand known piRNAs from human and mouse sperm samples (Krawetz et al., 2011; Hammoud et al., 2014). The role, if any, of piRNAs in mature sperm is currently unknown. It is also not known whether mature sperm piRNAs are intact and still bound to functional PIWI proteins and whether they have any role in transcriptional or post-transcriptional regulation in the early embryo.

Mature sperm cells contain a plethora of other small RNAs that we currently know little about. There are tRNA fragments that are 30–34 nt long, i.e., the size of piRNAs (Peng et al., 2012), small RNAs processed from piRNA clusters that are 20–21 nt long, (instead of the expected ∼30 nt of piRNAs in late spermatogenesis; Kawano et al., 2012) and fragments of repeats (Krawetz et al., 2011). Short transcripts derived from LINE-1 elements were recently found to positively regulate expression of LINE-1 repeats in early mouse embryos (Fadloun et al., 2013), so it is possible that among these fragments there are functional regulatory RNAs. Last, RNA molecules themselves (e.g., tRNAs) can carry modifications (Torres et al., 2014) that have been postulated to carry epigenetic information from father to offspring (Kiani et al., 2013).

## SPERM CHROMATIN

Sperm chromatin is highly specialized and is the end product of a highly complex differentiation program during which an impressive number of different testis-specific histone variants, histone-to-protamine transition proteins, and protamine genes are expressed. The role of many of these histone variants and histone-like proteins on gene expression during sperm differentiation and on mature sperm chromatin organization remains to be worked out. Post-fertilization, protamines are released from the paternal genome and replaced by maternal histones [for extensive reviews on protamines see (Lewis et al., 2003; Oliva, 2006; Balhorn, 2007; Rathke et al., 2014)].

In humans, 4–15% of the genome retains histones in sperm (Gatewood et al., 1987; Hammoud et al., 2009). Since the late eighties, it has been known that sperm nucleosomes are not randomly distributed along the genome (Gatewood et al., 1987). Comparing chromatin organization at the globin and protamine genes in sperm samples from different individuals, Gardiner-Garden and colleagues found that it is conserved between individuals (Gardiner-Garden et al., 1998). Interestingly, they also noted that some genes expressed early in development are packaged in nucleosomes while others expressed later are packaged in protamine toroids. Based on these, it was proposed that nucleosomes retained in sperm likely have a structural or regulatory role in late spermiogenesis and/or early embryo development.

Since 2009, several genome-wide sperm nucleosome profiles have been generated (Arpanahi et al., 2009; Hammoud et al., 2009; Brykczynska et al., 2010; Carone et al., 2014; Erkek et al., 2014; Samans et al., 2014). These confirmed that indeed the sites that remain packaged in nucleosomes are not randomly distributed along the genome. The first two studies (Arpanahi et al., 2009; Hammoud et al., 2009) showed that sperm nucleosomes are highly enriched at regulatory regions and in particular overrepresented at genes that regulate embryonic development such as the HOX genes (Hammoud et al., 2009; **Figures 2C,D**). This result is in agreement with the pre-existing notion that histones in sperm facilitate transcription regulation in the early embryo (Gatewood et al., 1987).

The availability of genome-wide profiles of histone enriched DNA in sperm made it possible to begin to dissect the mechanisms that determine which sites remain packaged by histones and which ones are replaced by protamines (Vavouri and Lehner, 2011; Erkek et al., 2014). Promoters of housekeeping genes and developmental regulators were found to overlap CpG islands, regions with high GC and CpG-content (reviewed in Deaton and Bird, 2011). Indeed, on a genome-wide scale and considering the non-repetitive parts of the genome, that pose problems when dealing with mapping sequenced reads, GC-content showed very strong correlation with histone retention in sperm (Vavouri and Lehner, 2011). This would suggest that the mechanism of nucleosome retention in sperm is tightly associated with sequence composition. Importantly, GC-content was more recently also confirmed to be strongly associated with histone retention in mouse sperm (Erkek et al., 2014). Considering all possible dinucleotides, Erkek et al. (2014) further found that, in mouse, it is CpG-dinucleotide composition that correlates best with the sites that retain histones in sperm. Also, according to both Hammoud et al. (2009) and Erkek et al. (2014), sites that retain histones in sperm are in general hypomethylated, however, it is unclear at this point whether this is due to a direct mechanistic link between DNA methylation and histone retention or whether they simply co-occur at CpG-rich regions.

Surprisingly, the two datasets published in 2014 show very different nucleosome distribution in human and mouse sperm (Carone et al., 2014; Samans et al., 2014). They show nucleosomes preferentially enriched at gene-poor/repeat-rich regions of the genome. Clearly, the six currently available genome-wide datasets of human and mouse sperm nucleosomes cannot all reflect the chromatin structure of sperm. Most likely, there is a critical step in sperm chromatin preparation and even slight variations in the protocol lead to isolation of very different fractions of the genome. According to Carone et al. (2014), this crucial step is the concentration of micrococcal nuclease. However, Samans et al. (2014) apparently used the protocol of Hammoud et al. (2009) but got the opposite results. A systematic comparison of the different sperm nucleosome isolation protocols and comparative analysis of the resulting data remains to be done to convincingly show what is really the organization of retained nucleosomes in mature sperm.

Sperm histones, like somatic histones, carry posttranslational modifications. Of particular interest, due to their important role in normal development and link with the maintenance of transcription patterns are the trithorax mark histone H3 lysine 4 trimethylation (H3K4me3) and the polycomb mark histone H3 lysine 27 trimethylation (H3K27me3). Sperm chromatin contains both of these (Hammoud et al., 2009; Brykczynska et al., 2010; Erkek et al., 2014). H3K4me3 is enriched at promoters of highly expressed genes during spermatogenesis (Hammoud et al., 2009; Brykczynska et al., 2010). It has also been reported that H3K4me3 marks some of the HOX cluster genes and paternally expressed imprinted genes (Hammoud et al., 2009). H3K27me3 marks primarily developmental regulators such as the HOX genes (Hammoud et al., 2009).

The genome-wide profiles of two histone variants are currently available for sperm. The histone variant H2AZ, which is associated with active regulatory regions in somatic cells, is limited to pericentric heterochromatin in mature sperm (Hammoud et al., 2009). H2AZ is, however, present at promoters of expressed genes in round spermatids (Soboleva et al., 2012; Hammoud et al., 2014). Since (according to the data from Hammoud et al., 2009 and Erkek et al., 2014) many promoters retain nucleosomes in sperm, it is unclear whether H2AZ-containing nucleosomes are lost from promoters in elongating spermatids before the histone-to-protamine transition, or whether nucleosomes lacking this histone variant replace existing nucleosomes during the histone-to-protamine transition. Unlike H2AZ, the histone variant H3.3 is found at expressed genes in round spermatids and is retained at the same promoters in mature sperm (Erkek et al., 2014).

Paternal histones can still be found in the zygote several hours post-fertilization in both human and mouse (van der Heijden et al., 2006, 2008; Puschendorf et al., 2008). To what extend and how exactly paternal histones contribute to chromatin organization and gene expression in the early embryo is not yet clear.

## SPERM DNA METHYLATION

Most of the genome of mature sperm is highly methylated (Molaro et al., 2011). This is in stark contrast to the globally lowly methylated oocytes and early embryos (Smallwood et al., 2011; Smith et al., 2012). However, CpG islands including those overlapping developmental regulators such as the HOX genes are hypomethylated (Hammoud et al., 2009; **Figure 2**). In contrast, promoters of key pluripotency regulators such as those of Oct4 and Nanog are highly methylated in human sperm (Hammoud et al., 2009). In light of these results, the relationship between DNA methylation in sperm and timing of expression in the early embryo is unclear.

The male germline goes through two waves of nearly complete DNA methylation erasure. One of these happens in the zygote, shortly after fertilization. At this stage, DNA methylation is erased specifically from the paternal genome (Oswald et al., 2000; Smith et al., 2012), affecting the majority of the genome but sparing paternal imprints (Edwards and Ferguson-Smith, 2007; Hajkova, 2011; Smith et al., 2012; Hackett and Surani, 2013). This ensures that DNA methylation gained by germ cells during the lifetime of the father is removed before the embryo starts development (Hajkova et al., 2002).

A small number of highly methylated regions, mostly associated with repeats, do nonetheless escape DNA methylation reprogramming. The most prominent example is the mouse IAP family of repeats (Howlett and Reik, 1991; Morgan et al., 1999; Lane et al., 2003; Kim et al., 2004; Guibert et al., 2012; Seisenberger et al., 2012). The mechanism that allows IAPs to evade DNA demethylation is currently unknown.

In search for molecular carriers of non-genetic information from father to offspring, DNA methylation analyses of sperm cells have featured prominently. At least in one study, changes in DNA methylation in sperm did indeed correlate with inheritance of a phenotype (Martinez et al., 2014), although the DNA methylation variation detected in sperm from different fathers was small and could be downstream of the cause of transmission of the phenotype. Not surprisingly, the strongest evidence of DNA methylation variation in sperm influencing phenotypic variation in offspring is related to IAP elements in mice (Morgan et al., 1999; Rakyan et al., 2003; Blewitt et al., 2006).

## CHALLENGES AND OPPORTUNITIES LYING AHEAD

Analyzing the sperm transcriptome poses several experimental and computational challenges. The first challenge is that sperm cells have very little RNA. It has been estimated that there are only 10–100 fg of total RNA per human sperm cell (Pessot et al., 1989; Krawetz, 2005), which is much less than that in somatic cells. Consequently, contamination of a sperm sample by somatic cells can heavily bias the resulting RNA profile. The second challenge is the absence of intact ribosomal RNA (Johnson et al., 2011; Goodrich et al., 2013). Quality metrics based on the “intactness” of ribosomal RNA (used for somatic samples) do not apply - although they could be used to assess somatic cell contamination. The third challenge is at the analysis stage. Transcript abundance quantification assumes that transcripts are intact. However, in sperm samples, only a tiny fraction (if any) of sequenced reads mapping to a gene correspond to intact transcripts. The mechanisms and dynamics of sperm transcript fragmentation/degradation are unknown. Until we have a better understanding of these processes and a systematic assessment of how accurately different gene expression quantification methods perform on sperm samples, we need to be cautious interpreting apparent abundance differences between different genes in the same sample and between samples. Transcript fragments also complicate the analysis of small RNAs. Degradation intermediates of ribosomal, mRNA and other transcripts largely outnumber sequence reads mapping to microRNAs. Although these reads can easily be identified and excluded, they also consume a very large proportion of the sequenced reads. So, if somatic small RNA samples can be profiled with as few as 5 million reads, sperm samples require several fold higher numbers of reads to achieve comparable depth of known regulatory small non-coding RNAs.

Analyzing sperm chromatin also poses great challenges. Because it is extremely compacted by protamines instead of histones one needs to use modified micrococcal nuclease digestion or chromatin immunoprecipitation protocols (e.g., Hammoud et al., 2009; Hisano et al., 2013; Carone et al., 2014). And because the different experimental protocols for protamine-compacted genomes have been less extensively used than those for histone-compacted genomes, their biases are also less understood. For example, as mentioned above, the recent genome-wide profiles of human and mouse sperm nucleosomes arrived to contradicting conclusions (Hammoud et al., 2009; Brykczynska et al., 2010; Hisano et al., 2013; Carone et al., 2014; Samans et al., 2014).

The most fundamental question regarding the transcriptome, chromatin and DNA methylation of sperm is whether they can transmit information about the father’s environmental exposures to the offspring. There are currently many reported cases of epigenetic inheritance via sperm (reviewed in Rando, 2012). For example, the father’s diet and traumatic experiences in early life seem to influence the phenotype of the offspring (Anderson et al., 2006; Carone et al., 2010; Ng et al., 2010; Gapp et al., 2014; Martinez et al., 2014). Although in some cases candidate carriers of this information have been identified (e.g., RNA or DNA methylation), the mechanisms are far from being adequately understood. Until mechanisms of epigenetic inheritance from father to offspring have been worked out and genetic inheritance has been definitively ruled out, it will remain questionable whether trans- and inter-generational epigenetic inheritance of phenotypes indeed exists in mammals (Heard and Martienssen, 2014).

## CONCLUSION

Although small, transcriptionally inert, with extremely compacted genome and virtually no cytoplasm, the sperm cell contains a plethora of small RNAs, a large number of DNA sequences packaged by histones and a distinctive DNA methylation profile. Until recently, the main purpose for studying the RNA, chromatin and DNA methylation of sperm (other than scientific curiosity for this highly peculiar cell type) was to identify potential biomarkers of male infertility. Today, there is an additional focus and this is to understand whether any of these “epigenomes” can transmit information from father to offspring. Therefore, it is now even more important to understand what information these epigenomes contain, how they are set, how they vary between individuals as well as between individual sperm cells, whether they are delivered to the egg upon fertilization and whether they have any impact on the development of the embryo and the phenotype of the offspring. During the past 5 years impressive advances have been made in describing the non-genetic contents of human sperm. Great opportunities are now lying ahead to also understand the mechanisms that set them and whether (and how) they influence gene and genome regulation in the early embryo.

## Conflict of Interest Statement

The authors declare that the research was conducted in the absence of any commercial or financial relationships that could be construed as a potential conflict of interest.
